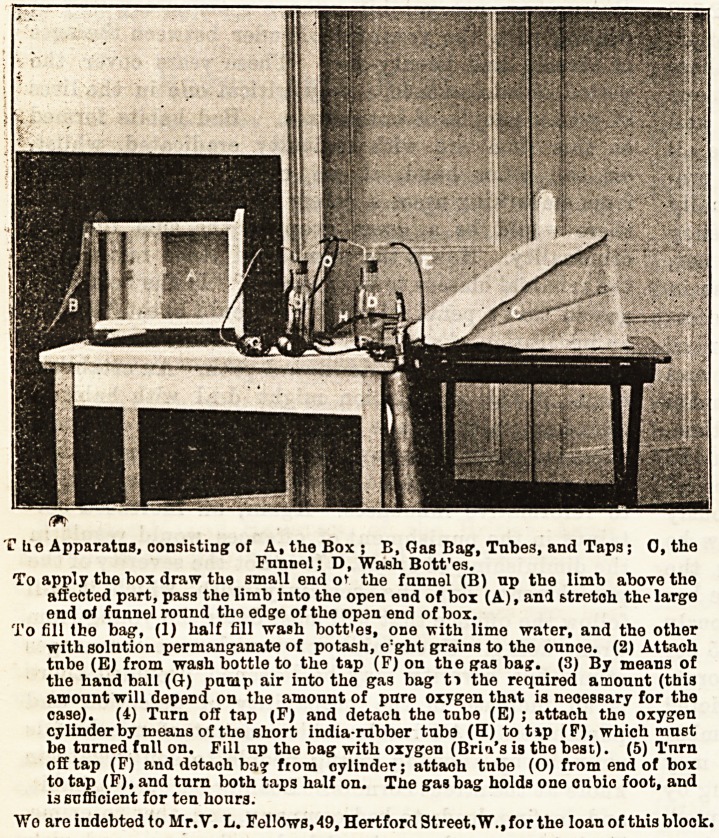# The Treatment of Ulcers, Wounds, and Other Surgical Cases by Oxygen

**Published:** 1896-07-04

**Authors:** George Stoker

**Affiliations:** Physician to the London Throat Hospital


### Medical Progress and Hospital Clinics.

*[The Editor will be glad to receive offers of co-operation and contributions from members of the profession. All letters should be addressed to* The Editor, at the Office, 428, Strand, London, W.C.]

### THE TREATMENT OF ULCERS, WOUNDS, AND OTHER SURGICAL CASES BY OXYGEN.

By George Stoker, M.R.C.P.I., Physician to the London Throat Hospital.

This treatment has advanced far beyond the limits that were at first anticipated. Like all good and useful discoveries, it has not stood still, but has proved itself capable of development in several directions. The treatment consists in the exposure of the affected part to oxygen gas; this exposure may be either continuous or intermittent. The system in its simplest form is seen in the treatment of a hand or arm, or foot, and leg below the knee; for any of these the apparatus is practically the same.

I always use Brin's oxygen, which can be obtained in cylinders holding from ten feet upwards. I recommend anyone using these cylinders to get a "fine adjustment valve," to be attached to the cylinder, as it much facilitates decanting the oxygen into the bag, and avoids waste. Having enclosed the limb in the box and filled the bag, the next step is to attach the bag to the box and turn on the taps, and the treatment is commenced. One bag is sufficient under ordinary circumstances for twelve hours. The amount of pure oxygen used varies according to the case. In recent cases of wounds, burns, &c., it is sufficient to use a quarter of a bag of pure oxygen and three-quarters of purified air, but in chronic cases, equal parts of air and oxygen is best. To measure the oxygen accurately, it is necessary to have a bag containing one quarter of a cubic foot; in "The Oxygen Home" we have a gasometer, but this is manifestly impossible except in institutions, and a quarter of a foot bag will be found sufficient and convenient.

The Apparatus, consisting of A, the Box; B, Gas Bag, Tubes, and Taps; C, the Funnel; D, Wash Bott'es.

To apply the box draw the small end of the funnel (B) up the limb above the affected part, pass the limb into the open end of box (A), and stretch the large end of funnel round the edge of the open end of box.

To fill the bag, (1) half fill wash bott'es, one with lime water, and the other with solution permanganate of potash, eight grains to the ounce. (2) Attach tube (E) from wash bottle to the tap (F) on the gas bag. (3) By means of the hand ball (G) pump air into the gas bag to the required amount (this amount will depend on the amount of pure oxygen that is necessary for the case). (4) Turn off tap (F) and detach the tube (E); attach the oxygen cylinder by means of the short india-rubber tube (H) to tap (F), which must be turned full on. Fill up the bag with oxygen (Brin's is the best). (5) Turn off tap (F) and detach bag from cylinder; attach tube (O) from end of box to tap (F), and turn both taps half on. The gas bag holds one cubic foot, and is sufficient for ten hours.

We are indebted to Mr. V. L. Fellows, 49, Hertford Street, W., for the loan of this block.

Beyond what I have stated, there are at present no data for exactly determining the best strength of oxygen to use in any particular case. My present belief is that such exact data may possibly be found in the state of the micro-organisms in the wound, and I am making careful investigations in this direction, and will publish the results later on.

The wounds should be cleansed twice daily with warm water. The oxygen causes the formation of a parchment-like film round the margins of the wound over the newly-growing skin, and unless this film is removed it prevents the oxygen getting at the parts underlying it, and delays healing; its removal is best effected with fine dissecting forceps.

It rarely occurs that after some time, and when only a very small part of the wound or ulcer remains unhealed, the oxygen seems to lose its effect and the wound remains stationary. Under these circumstances it is best to leave off the oxygen, and apply a little simple water dressing. This result is not by any means common, and I am at present unable to offer any reason for its occurrence.

I have found the treatment most successful in cases of ozœna and suppurative middle-ear disease. There the application is very simple. Only the bag tube and taps, and the wash-bottles are required. The bag is half filled with oxygen, and filled up in the usual way with purified air. A small ear or nose piece is fitted in on the end of the tube. If the ear is to be treated the ear-piece is passed into the external auditory meatus and the tap turned on. The oxygen should be applied about three to four hours daily for periods of half an hour at a time. The ears should be syringed with warm water before each application of oxygen.

For ozœna the same strength of oxygen should be used as in ear cases. The nose piece should be passed into one nostril and the other plugged with cotton wool. This compels the patient to breathe through the mouth and leaves the oxygen more or less in undisturbed contact with the nose or naso-pharynx and prevents the patient inhaling too much oxygen, which induces headache.

In ozœna cases the oxygen should be continued for half to one hour four or five times daily, the nose being cleansed and thoroughly freed from scabs and crusts before each application of oxygen.

The oxygen treatment is further being applied to diseases of the antrum, eye, uterus, &c., and is, indeed applicable wherever there is an ulcerated or suppurating surface. Its advantages may be thus summarised: It heals cases that have resisted other forms of treatment; it heals in far less time than any other system (this is especially marked in recent cases); it relieves pain, stops foul discharges, and bad smells; it forms a healthy and vascular cicatrice, which in cases healed many months ago has shown no tendency to break down.

I have made many careful cultivations from wounds and ulcers rapidly healing under oxygen treatment, and have in all found micro-organisms staphylococci aureus and albus, in abundance. I am inclined to believe that these bodies absorb poisonous matters from the wounds, &c., on which they live, and in turn their excreta poisons the same wound; but that oxygen oxidises and purifies these excreta and thus assists healing. It is also plain that oxygen acts as a stimulant, and induces a flow of blood to the part, and that this blood is directly oxygenated.

## Figures and Tables

**Figure f1:**